# Investigation of the Temperature and Horizontal Freezing Force of Loess in Three-Dimensional Freezing

**DOI:** 10.3390/ma17184614

**Published:** 2024-09-20

**Authors:** Yidan Yin, Fei Liu, Dongqi Tang, Longze Chen, Binbin Yang

**Affiliations:** 1 School of Resources and Environment, Xi’an University of Science and Technology, Xi’an 710054, China; 22209226048@stu.xust.edu.cn (Y.Y.); geoloessology@xust.edu.cn (F.L.); 2 School of Civil Engineering, Xuchang University, Xuchang 461002, China; tdq_tm@xcu.edu.cn; 3 School of Electronic Science and Engineering, University of Electronic Science and Technology of China, Chengdu 611731, China; a2673496757@outlook.com

**Keywords:** loess, three-dimensional freezing test, horizontal freezing force, temperature

## Abstract

Seasonal frozen soil has significant impacts on changes in soil mechanical properties, settlement, and damage to foundations. In order to study variations in the temperature and horizontal freezing force of loess during three-dimensional freezing, a three-dimensional freezing model test of loess was carried out. This experiment analyzed and studied the soil temperature change distribution characteristics, horizontal freezing force distribution rules, and water migration phenomena caused by temperature. The research results show that the temperature change in soil samples exhibits a “ring-like” decrease from the outside to the inside. When the soil temperature reaches the supercooling point, the cooling curve jumps and rises, and this is accompanied by a stable section with constant temperature. In the late freezing period, the temperature rate drops slowly. Under the action of freezing, the horizontal freezing forces at different positions have similar change characteristics and can be divided into four change stages: stable stage, rapid freezing stage, “secondary” freezing stage, and freezing–shrinkage–rebound stable stage. At lower moisture contents, loess samples undergo freeze–thaw shrinkage during the freezing process. During the rapid freezing stage of soil samples, the water in the soil sample migrates and causes secondary freezing. After the rapid freezing stage, the soil temperature continues to decrease, and the horizontal freezing force no longer decreases.

## 1. Introduction

Frost damage to shallowly buried buildings in permafrost regions is caused by uneven deformation, horizontal freezing forces, and normal and tangential freezing forces. Among them, the horizontal freezing force is the main cause of frost damage to shallowly buried buildings [1]. The Qinghai Tibet Plateau and Northwest China are the main seasonal frozen soil distribution areas of loess. Engineering construction in these areas has caused a lot of loess frost damage due to seasonal low-temperature freezing. The freezing force causes cracks on the highway surface, slope sliding, roadbed elevation, and damage to retaining structures and water channels. Therefore, the evolution of the horizontal freezing force of loess during the freeze–thaw process is an important factor that cannot be ignored in geological disaster prevention and control [2].

Many scholars have conducted investigations on the temperature changes in frozen soil during the soil freezing process. In the frozen soil area, the periodic change in temperature causes the expansion and contraction deformation of the soil, changing its physical and mechanical properties [3]. Temperature is the main driving force for soil moisture migration and freezing [4]. During the freezing process, the phase transition of water in the soil changes the pore structure, which is the microscopic cause of macroscopic freezing and deformation of the soil. The freezing of the surface soil causes a decrease in the liquid water content in the soil and an increase in the matric suction of soil particles. Lai et al. [5] conducted a one-way freezing test of an open system without pressure replenishment and found that when the soil reaches the freezing point, the pore water rapidly transforms into ice, causing negative pressure on the pore water and causing water to migrate to the freezing zone. When the soil temperature T_s_ decreases to a certain value, the water in the soil begins to freeze, and the temperature rises rapidly to a stable state, which is the supercooling phenomenon, and T_s_ is the supercooling point. The presence of pore water will cause the freezing point to drop and result in freezing deformation [6], and the soil will enter a supercooling stage before it can further transform into a spontaneous process, so the freezing temperature and supercooling temperature directly affect the freezing characteristics of the soil [7]. Wu et al. [8] found that freeze–thaw cycles can cause step deformation of loess slopes and change the distribution of moisture, with the surface moisture content being greater than the bottom. Xiao et al. [9] believe that the soil freezing process leads to the occurrence of supercooling, which delays the formation of ice and keeps the unfrozen water constant. The soil will continue to freeze only when the soil temperature falls below the freezing temperature.

During the soil freezing process, the moisture in the soil freezes into ice. The migration of liquid causes deformation of the soil [10,11,12], thus generating a freezing force. The content of unfrozen water in frozen soil is related to the temperature, and unfrozen water in frozen soil is the source of liquid water migration. The properties of frozen soil also change with the variation in unfrozen water content [13,14]. Harlan et al. [15] proposed a fluid dynamics model under the coupling effect of heat and mass migration and water migration in partially frozen soil and studied the redistribution of soil water and the permeability of frozen soil. Zhou et al. [16] conducted unidirectional freezing tests on large mud clay columns and found that moisture migration is related to the temperature and the pore structure. The temperature and pore structure provided the driving force and transport channels for moisture migration, and moisture migration changed the thermal balance and pore structure of the soil column. Ma et al. [17] conducted a unidirectional freezing test on coarse-grained saline soil with local fine sand accumulation and studied its moisture migration law and deformation characteristics. The results showed that compared with natural gradation, the moisture migration and deformation of the samples in the local fine sand accumulation layer were more severe. Gao et al. [18] conducted a unidirectional freezing test on fine-grained soil and found that when the water content was low, the effect of water migration on freezing deformation was more obvious.

Existing scholars have also investigated the evolution of freezing forces under unidirectional freezing soil conditions. The freezing force of the soil during freezing is mainly caused by the migration of water under hydraulic gradients and thermal gradients [19]. Ji et al. [20] conducted one-dimensional freezing tests on silty clay under different constrained stiffness conditions and studied the evolution law of FHIP (frost-heave-induced pressure) during the hydrothermal coupling process. The study found that FHIP was affected by the constraint stiffness. The increase in constraint stiffness led to an increase in deformation and an increase in the FHIP. Hao et al. [21] studied the evolution of FHIP based on one-dimensional freezing experiments and proposed a prediction method for the maximum FHIP. The study pointed out that the evolution of FHIP is affected by the movement of the ice layer. When water stops migrating and ice stops growing, the FHIP also stops increasing. Tong et al. [22] obtained the general law of horizontal frost-heave force of soil around the pile during the freezing process through indoor experiments. Zhang et al. [23] used a self-designed tangential freezing force measurement device to study the evolution of tangential freezing force of clay silt in a unidirectional freezing model test and analyzed the effects of soil temperature and moisture content on the tangential freezing force.

The purpose of this paper is to understand the distribution and evolution of freezing forces at different positions of soil samples during the freezing process and to analyze the influence of temperature on the distribution of the freezing force. This article takes loess in seasonal frozen soil areas as the research object and uses the indoor model test method to study the changes in soil temperature and horizontal freezing force during the three-dimensional freezing process of loess; from this, the soil temperature time curve and horizontal freezing force time history curve are drawn. By combining its temperature and freezing force, the influence of temperature on the generation and evolution of the freezing force of loess in a three-dimensional freezing environment are explored. This article provides effective measures and methods for managing the collapse and landslide of loess slopes in Xianyang area. 

## 2. Materials and Methods

**Figure 1:**
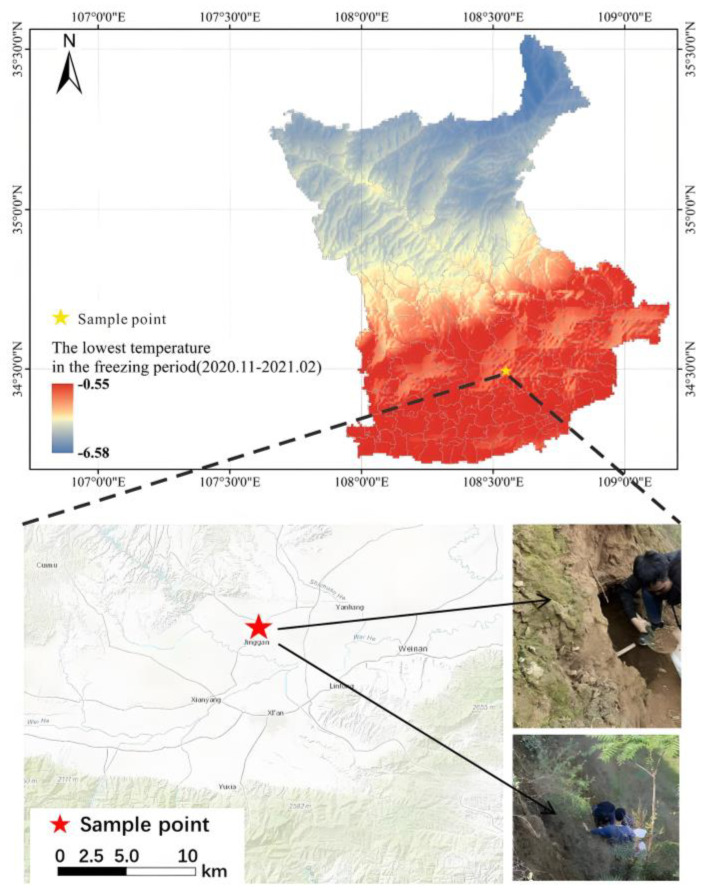
Investigation area and sampling points.

As shown in Figure 1, the sampling point (N34° 29′ 26″ N, E108° 45′ 41″ E) is located in Miaodian Village, Jingyang County, Xianyang City, Shaanxi Province.

Shaanxi Province is located in the middle reaches of the Yellow River in northwest China and has a continental monsoon climate. The annual and daily temperature ranges are relatively large, and the extreme temperature difference between winter and summer is even greater. The precipitation distribution is uneven, and precipitation in winter is less than that in summer. The cold air in winter comes from the high-latitude continental area, which is cold and dry. There are large areas of loess distributed in the province, and the loess in this region is located in the seasonally frozen soil area. The annual average temperature in Jingyang County, Xianyang City, is 13 °C. The freezing period in this area is from November to February each year. The lowest temperature can reach −14 °C, and the annual average precipitation is 548.7 mm. In this area, the loess foundation is limited by the load of building structures, which will cause uneven lifting of the foundation and cracking of the upper structure, leading to damage to building structures. In winter, the moisture in the roadbed freezes into ice, causing the roadbed to rise and produce longitudinal and transverse cracks, and even bulging in areas with severe local deformation. It can be seen that loess geological disasters caused by freezing cannot be ignored in engineering construction.

The sampling time was in mid-October 2021, and samples were taken from a natural slope within a local landslide control project site. The vegetation at the field observation sampling point was sparse, and the soil surface structure was loose. The original loess was taken at about 3 m from the top of the slope. Before sampling, the site was first leveled, the weeds on the surface removed, and root soil with a high surface moisture content was dug out. After cleaning horizontally inward, the original soil was taken horizontally. At the same time, a ring knife sample was taken to determine the basic physical parameters of the loess. The original loess retrieved from the site was air-dried and passed through a 2 mm sieve. The natural density, natural moisture content, and air-dried moisture content of the loess were determined by the ring knife method and drying method, and the dry density of the loess was calculated. The liquid limit and plastic limit were determined by the combined determination method of liquid and plastic limits, and the liquid index and plasticity index of the loess were calculated. The optimal moisture content and maximum dry density were determined by the compaction test. The basic physical parameters are shown in Table 1. It can be seen from the table that the loess in this area belongs to silty clay.

**Figure 2:**
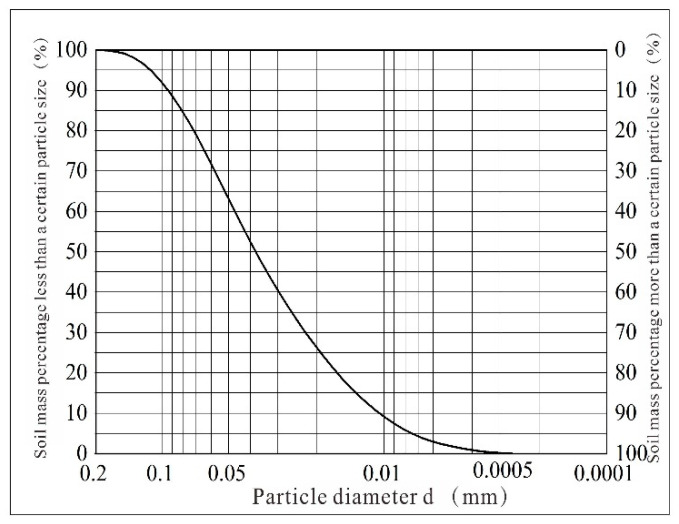
Grain-size distribution curve of the loess.

To measure the particle size, a Bettersize 2600 laser particle size analyzer (Dandong, China)was used. As shown in Figure 2, loess is mainly composed of silt and clay particles, with silt accounting for 72.28% and clay accounting for 24.32%. Since the uniformity coefficient of loess Cu = 4.45 and the curvature coefficient Cc = 1, the loess grading is poor.

### 2.1. Experimental Details

The wet soil was prepared to obtain the optimal moisture content of 17.5% and the maximum dry density ρ_dmax_ = 1.7 g/cm^3^. The prepared wet soil was placed in a moisturizer and left to stand for 24 h to allow it to be fully mixed. Finally, the wet soil was made into samples with a diameter of 100 mm and a height of 50 mm. This experiment collected data on the temperature field and freezing force changes in the three-dimensional frozen model test of loess and analyzed the variation patterns of each variable and their relationships.

**Figure 3:**
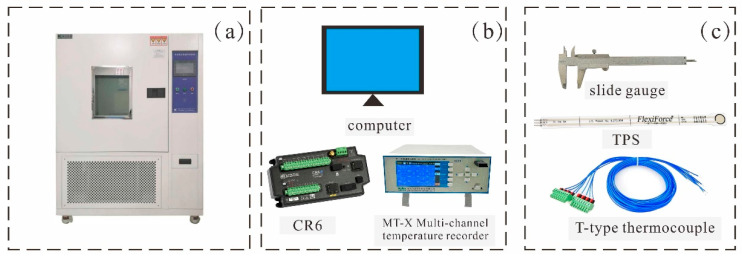
Experimental device diagram showing the (**a**) high- and low-temperature test chamber; (**b**) date acquisition device; and (**c**) monitoring device.

The test temperature is controlled by a high and low temperature alternating damp heat test chamber (Shanghai, China, BPHJ-060B). The monitoring system consists of a pressure monitoring device, a temperature monitoring device, a data acquisition device, and a vernier caliper (Figure 3).

The working temperature range of the high- and low-temperature alternating humidity test chamber is −40 °C to 150 °C, with temperature fluctuations of less than 2 °C. The internal dimensions are 400 × 500 × 500 mm (length × width × height). A 100 mm thick rock wool insulation layer is filled around the door of the test chamber, and there is a 50 mm diameter test hole in the middle of the side centerline for the entry and exit of external sensor circuits. The temperature monitoring device consists of a T-type thermocouple (probe length 1 cm, wire diameter 1 mm) and an MT-X multi-channel temperature recorder. The pressure monitoring device consists of a FlexiForce™-A201 single-point flexible film pressure sensor (TPS) and a CR6 data acquisition instrument, and a vernier caliper was used to record the changes in the diameter of the loess sample during the test.

The temperature control is based on the annual temperature changes in the Xianyang area. The lowest temperature during the freeze–thaw period is −9 °C, while the highest temperature is 21 °C, and the average temperature is 5 °C. As the shallow surface soil undergoes seasonal freeze–thaw processes, the temperature shows periodic changes. Its temporal variation can be described by a sine function [24]. During the freeze–thaw process, 20 °C and −10 °C are taken as the upper and lower limits of the temperature of the loess sample, and the cooling rate is set to 0.25 °C/min. Its temporal variation is shown in Equation (1).

(1) $$y=15\sin\left(\frac{\pi x}{12}+\frac{\pi }{2}\right)+5\;\left(x\in \left[0,12\right]\right),$$

In the formula, *y* is the temperature (°C), and *x* is the time (h).

**Figure 4:**
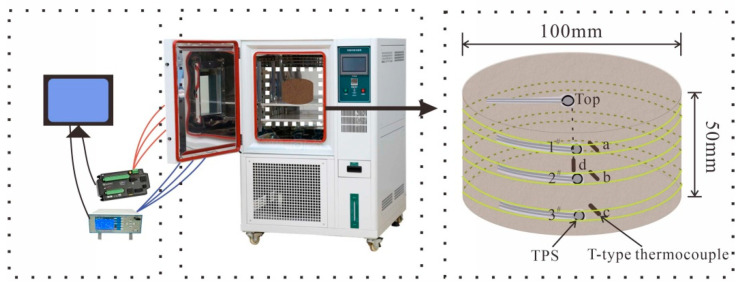
Test instrument connection and sensor pasting method.

The monitoring system measures the temperature and freezing force of loess samples using T-type thermocouples and a TPS, respectively. The T-type thermocouple is inserted into the side of the loess sample at a height of 10 mm, 25 mm, and 40 mm, a depth of 1 mm, and at the center of the loess sample (respectively marked as a, b, c, and d). The TPS was attached next to T-type thermocouples a, b, and c, (denoted as 1^#^, 2^#^, and 3^#^ from top to bottom) and the TPS is attached to the top surface, as shown in Figure 4. The surface of the loess sample with the sensor arranged was wrapped with a layer of plastic wrap to prevent the moisture content of the loess sample from decreasing during the freezing process. The position of the sensor was fixed with a rubber band to prevent experimental errors caused by poor sensor contact, and it was then placed in a high- and low-temperature alternating humidity and heat test chamber. The TPS was attached to the side of a steel sheet with the same diameter as the loess soil sample, and experiments were carried out simultaneously with the experimental group to eliminate experimental errors caused by the influence of temperature changes on the TPS. A T-type thermocouple was placed near the soil sample to monitor the ambient temperature of the test chamber to prevent loss and hysteresis during the heat exchange between the test chamber and the soil sample. The TPS and T-type thermocouples were connected to the CR6 data acquisition instrument and the MT-X multi-channel temperature recorder, respectively, and these were connected to a computer to control the data acquisition. During the experiment, one data point was collected every 10 s to monitor the temperature and horizontal freezing force of the soil sample. A vernier caliper was used to record the diameter changes in loess samples 1^#^, 2^#^, and 3^#^ during the freezing process. The model test apparatus is shown in Figure 4.

### 2.2. TPS Calibration Curve Test

**Figure 5:**
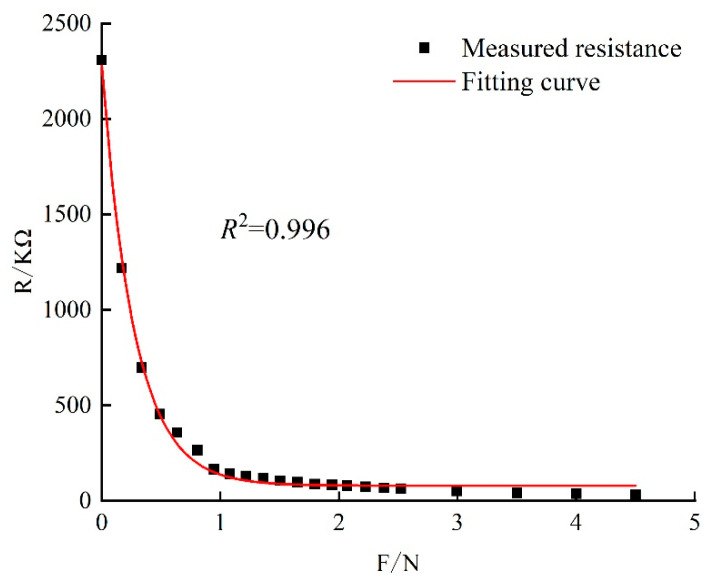
TPS (D100 mm) calibration curve.

Calibration is required before using the TPS. Liu et al. [25] believe that curvature has a certain impact on the TPS and conclude that when the curvature reaches a certain size, the TPS needs to be recalibrated. In order to reduce the error of the experiment, the FlexiForce™-A201 single-point flexible film pressure sensor was calibrated on a rigid cylinder with a diameter of 100 mm and a curvature of 0.2 (1/cm) using the double-load calibration method [26] to obtain a pressure-resistance curve with a curvature of 0.2, as shown in Figure 5.

The TPS calibration curve is fitted by an exponential function, and the fitting formula is the TPS calibration formula, shown in Equation (2). Based on the TPS calibration formula and in combination with the program arithmetic in the CR6 data collector, the evolution of the freezing force during the freezing process can be obtained through the resistance change in the TPS.

(2) $$R=2199.08\times e^{-3.57F}+79.46,$$

In the formula, R = output resistance (KΩ), and F = load (N).

## 3. Results and Discussion

### 3.1. Temperature Variation Pattern

**Figure 6:**
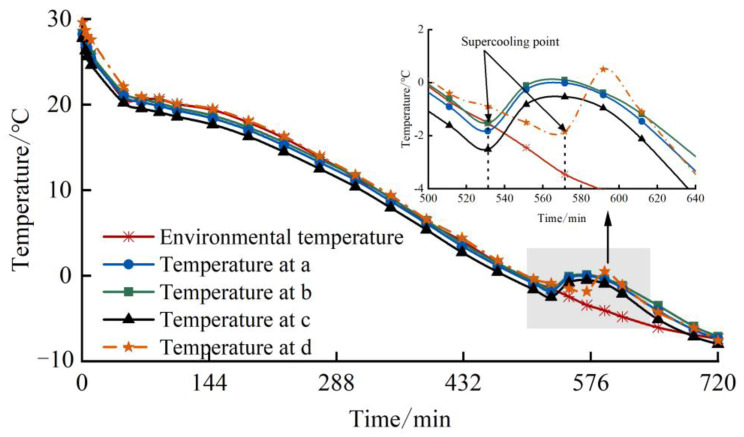
Temperature variation curves.

Figure 6 shows the temperature variation curves of loess samples at different heights during the experimental process.

As shown in the figure, the temperature in the center of the loess is 1 °C~2 °C higher than the temperature outside the loess sample. This is because the size difference between the test chamber and the soil sample is too large. Therefore, during the freezing test, there is a lag effect between the ambient temperature of the test chamber and the temperature of the soil sample, manifested as the surface temperature reaching the ambient temperature earlier than the internal temperature. In the early stage of freezing, due to the high temperature of the loess sample itself, the temperature of the soil decreases rapidly. When the ambient temperature reaches the preset temperature, the temperatures at points a and c are basically close to the ambient temperature because they are affected not only by the side temperature but also by the top and bottom surfaces. The temperature at point b is higher than that at points a and c. The hysteresis effect causes the temperature of point d to be higher than that of points a, b, and c. When the temperature of the sample drops to the supercooling point, the bound water in the loess sample begins to freeze and undergoes a phase change to form ice crystals, releasing a large amount of latent heat [27]. Under the three-dimensional freezing test, the surface loess particles begin to freeze and release latent heat, resulting in a small jump in temperature at points a, b, and c. After the water molecules in the surface soil freeze into ice, the free water molecules on the particle surface decrease and the matric suction increases, resulting in the unfrozen water inside the loess sample migrating to the outer side with the lower temperature and continuing to freeze into ice on the outer side. As ice crystals grow steadily, the temperature inside the soil sample begins to drop. From the figure, it can also be seen that due to the “barrier” effect of surrounding particles at the center point d, the hysteresis effect of the supercooling point at this location is significant. As the temperature continues to decrease to the termination temperature of the experiment, the rate of the temperature drop of the soil at each height slows down, which is a stable cooling stage. Prior to this, the free water, capillary water, and weakly bound water would have all frozen. Due to the strong adsorption force on the surface of soil particles [28], strongly bound water hardly changes phase. However, with the freezing of the soil, the thermal conductivity and specific heat capacity of the soil change, resulting in the heat exchange capacity between the loess sample and the environment remaining constant per unit of time [13].

### 3.2. Analysis of Freezing Force Test Results

During the freezing process of the sample, the unfrozen water content and ice content inside the soil affect the changes in the freezing characteristics of the soil. The water inside the loess sample will undergo phase transitions with temperature changes. These phase changes will cause the interaction forces between the soil particles and soil water inside the loess sample, resulting in the freezing force of the loess sample [29]. The generation of the freezing force is influenced by many factors, including the soil moisture content, temperature, and fine particle content. During the freezing process, loess samples are in a superposition of two states—frost heave and frost shrinkage—rather than a single state of frost heave or frost shrinkage [30,31]. Liu et al. [32] studied the effect of initial moisture content on the freezing deformation of cohesive soil. They found that when the soil saturation is greater than 0.75, the soil will expand; otherwise, the soil will shrink. Ma et al. [33] pointed out that when the moisture content of the soil is lower than the initial frost-heave moisture content, the soil will be deformed mainly by frost shrinkage and thaw expansion. The initial frost-heave moisture content of silty clay is approximately equal to the plastic limit. The moisture content of the experimental loess is 0.81 to 0.92 times its moisture content, while the experimental loess moisture content is 0.91 times its plastic limit moisture content. Therefore, theoretically, the deformation of reshaped loess during the freeze–thaw cycle is mainly due to freeze shrinkage and thaw expansion.

**Figure 7:**
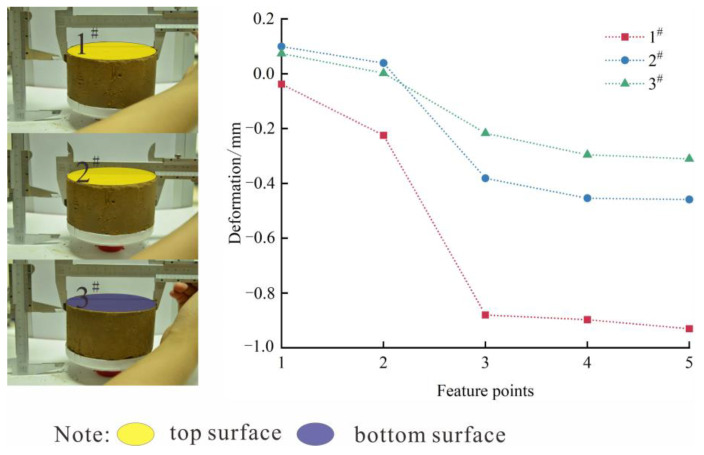
Deformation diagram of feature points.

In order to verify whether the loess sample undergoes freeze–thaw shrinkage deformation during the freezing process, this was verified by measurement. According to the temperature change curve, the characteristic points at 530 min, 550 min, 600 min, 650 min, and 720 min during the freezing process were recorded as 1, 2, 3, 4, and 5. A vernier caliper was used to measure the diameter changes at positions 1^#^, 2^#^, and 3^#^, as shown in Figure 7.

It can be seen from the figure that as the temperature decreases, the diameters at positions 1^#^, 2^#^, and 3^#^ of the loess sample decrease, and shrinkage deformation occurs. The largest freeze–thaw shrinkage deformation occurs at position 1^#^, followed by position 2^#^, and the smallest change occurs at position 3^#^. The uneven deformation of loess samples is mainly caused by their own weight. Under the action of their own weight, the loess at the bottom of the loess sample tends to move outward, increasing the friction between the soil particles. Therefore, the loess located in the middle and lower parts needs to overcome the influence of the self-weight of the upper loess during the freeze–thaw deformation stage. The maximum freezing force at the top is only −9.39 kPa, while the maximum horizontal freezing force at the side is −26 kPa. The freezing force at the top is 16.61 kPa lower than the maximum horizontal freezing force.

**Figure 8:**
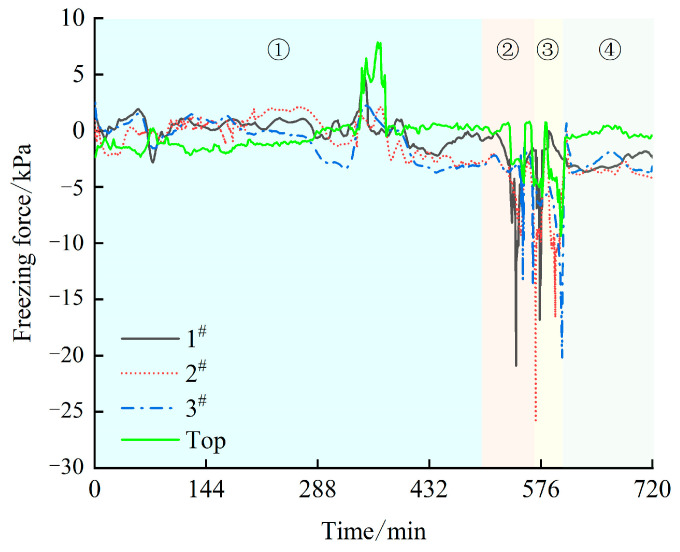
The variation curve of the horizontal freezing force.

The curve of the freezing force at different heights of the sample as a function of time is shown in Figure 8.

The curves of the freezing force at different heights of the sample over time are shown in Figure 8. As shown in the figure, when the temperature drops to the supercooling point, the soil begins to generate a freezing force. With the continuous development of ice crystals, the freezing force rapidly decreases. Due to the influence of water migration, the freezing force fluctuates to a certain extent. When the soil temperature is lower than the maximum freezing force temperature, the freezing force of the soil gradually increases. The maximum value of the freezing force on the top is only −9.39 kPa, while the maximum value of the horizontal freezing force on the side is −26 kPa. The freezing force on the top surface is 16.61 kPa less than the maximum value of the horizontal freezing force.

According to the trend in the curve changes, the changes in the freezing force during the freezing process can be divided into four stages: ① Stable stage: The soil temperature basically does not produce a freezing force until it reaches the supercooling point; ② Rapid freezing stage: When the soil temperature drops to the supercooling point, the freezing force suddenly decreases. When the soil begins to freeze, the ice crystals cannot fill the pores due to the loss of water and shrinkage of the soil particles. Ice crystals play a cohesive role between soil particles, and the diameter of the soil continuously decreases and the freezing force decreases, resulting in a peak. The freezing force peak varies at different heights, and the temperature of the upper and lower parts of the soil sample is lower than that of the middle part. Therefore, the freezing force of the upper and lower parts first reaches its peak. However, due to the influence of gravity on the loess sample itself, the soil sample at the bottom is not only affected by the inward freezing force but also by the outward gravity, so the freezing force is smaller than that of the upper part. ③ Secondary freezing stage: When the internal temperature of the loess sample reaches the supercooling point, the unfrozen water content inside begins to decrease, and the internal soil freezes. At this time, the freezing force at positions 1^#^, 2^#^, and 3^#^ is the original freezing force plus the freezing force generated by the internal freezing, and secondary freezing occurs. Since the migration of unfrozen water during the rapid freezing phase of 2^#^ leads to a decrease in the water content in the middle, the freezing force is the largest. ④ Freeze–thaw rebound stable stage: After the soil temperature drops below the maximum freezing force temperature, the overall freezing of the loess sample leads to a decrease in pore water pressure, and the freezing force of the soil gradually rebounds.

### 3.3. Effect of Temperature on Freezing Force

**Figure 9:**
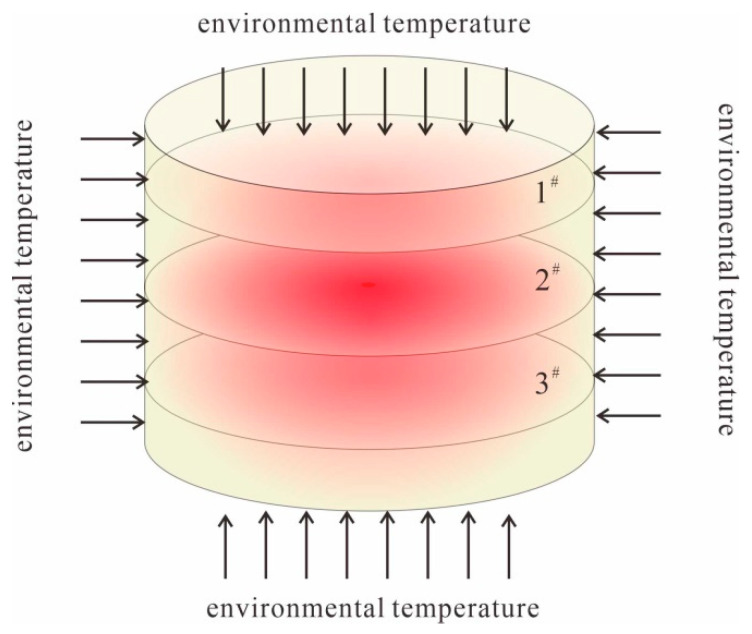
Schematic diagram of the “ring-like” temperature decrease.

**Figure 10:**
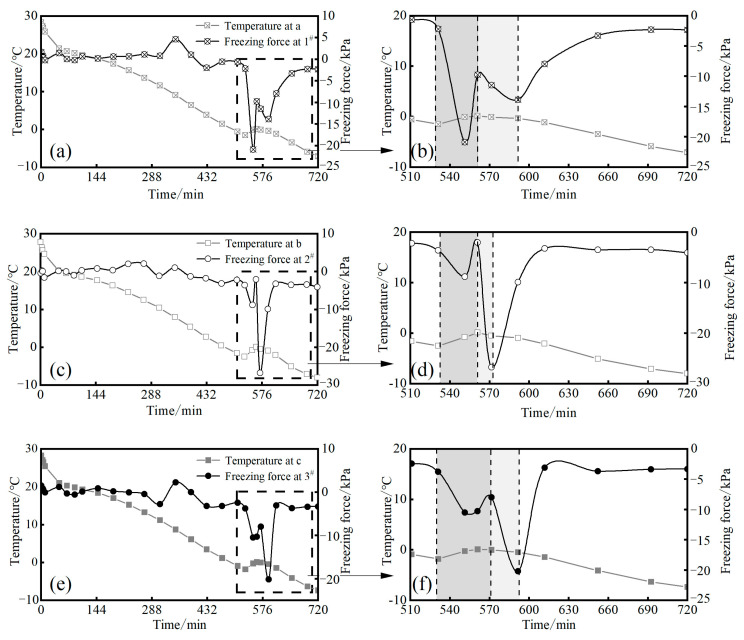
Curves of the horizontal freezing force with temperature variations.

As shown in Figure 9, under three-dimensional freezing conditions, when the soil temperature drops to the initial freezing temperature, the loess sample begins to freeze from the outside to the inside. The outer side freezes first, causing unfrozen water inside to migrate to the outer side, leading the loess sample to continuously freeze. This accounts for the high surface moisture content when the frozen soil melts with a rise in temperature. During the freezing process, the loess sample undergoes freeze–thaw shrinkage deformation, leading to the emergence of a horizontal freezing force on the sample’s side. As shown in Figure 10, the first peak of the freezing force occurs during the rapid freezing stage of the soil after the temperature reaches the supercooling point. Interfaces at positions 1# and 3# are more in contact with the external environment than those at position 2#, leading to the freezing phenomenon at the internal interfaces of positions 1# and 3#. The water within the soil sample freezes, but due to its low moisture content, it does not completely fill the interstices between the soil particles, leading to freeze-induced deformation. The soil immediately exhibits a negative freezing force, with the forces at positions 1# and 3# exceeding that at position 1#. As the environmental temperature continues to decrease, the internal temperature begins to freeze, accelerating the rate of the water and ice phase transition and water migration, resulting in a “secondary” freezing phenomenon in loess samples. The freezing force at position 2 # is coupled by internal and external freezing forces, resulting in a second peak. The maximum value of the horizontal freezing force is significantly influenced by the moisture content [34]. Due to the internal freezing of the soil sample, positions 1# and 3# also experience a “secondary” freezing peak as a result of the freezing shrinkage at the center.

## 4. Conclusions

This study takes a slope loess in Xianyang as the research object, and through indoor three-dimensional freezing model experiments, investigates the changes in the temperature field and freezing force of loess in the Xianyang area, exploring the influence of freezing on the temperature, deformation, and horizontal freezing force of loess slopes. It provides effective data for collapse prevention and risk avoidance warning in this area. It was found that the temperature changes on the surface of loess samples at the upper, middle, and lower positions were not significantly different in the three-dimensional freezing environment, while the internal temperature showed a hysteresis phenomenon. The temperature changes of the soil samples showed a “ring-like” decrease from the outside to the inside; when the soil temperature reaches the supercooling point, the cooling curve jumps and enters the later stage of freezing with a stable period of constant temperature, and the temperature rate decreases slowly.

In the same soil sample and under the same freezing action, the horizontal freezing force of loess samples at different heights has similar variation characteristics, which can be divided into the following four stages: stable stage, rapid freezing stage, secondary freezing stage, and freeze–thaw rebound stable stage. The soil sample in this experiment is silty clay, and its water content is 0.91 times the plastic limit, so the soil sample undergoes freeze–thaw deformation. However, due to the migration of water inside the soil, the degree of freeze–thaw shrinkage in each part is different, and the horizontal freezing force is also different.

The horizontal freezing force is not only affected by temperature but also by moisture migration. The horizontal freezing force begins to appear when the soil temperature is lower than the freezing temperature. As the loess sample freezes quickly, the moisture in the loess sample migrates and undergoes a secondary freezing. After the secondary freezing is completed, the soil temperature continues to decrease, and the horizontal freezing force no longer decreases.

## Figures and Tables

**Table 1:** Basic physical indicators of the loess.

Natural Water Content /%	Dry Density /g∙cm^−3^	Optimum Moisture Content /%	Maximum Dry Density/g∙cm^−3^	Liquid Limit %	Plastic Limit/%	Liquidity Index%	Plasticity Index %
14.9	1.59	17.5	1.7	29.6	18.6	−0.3	11.3

## Data Availability

All data generated or analyzed during this study are included in this published article.
